# Denosumab-associated jaw bone necrosis in cancer patients: retrospective descriptive case series study

**DOI:** 10.1186/s40902-023-00391-9

**Published:** 2023-06-30

**Authors:** Ji-Yeon Kang, Sang-Yup Kim, Jae-Seok Lim, Jwa-Young Kim, Ga-Youn Jin, Yeon-Jung Lee, Eun-Young Lee

**Affiliations:** 1 Department of Oral & Maxillofacial Surgery, College of Medicine, Chungnam National University, Moonhwa-ro 282, Jung-Gu, Daejeon, 35015 Korea; 2 Department of Oral & Maxillofacial Surgery, College of Medicine and Medical Research Institute Chungbuk, National University, Chungdae-ro 1, Seowon-Gu, Cheongju, 28644 Chungbuk Korea; 3 Department of Oral & Maxillofacial Surgery, Daejeon St. Mary’s Hospital, Daeheung-ro 64, Jung-Gu, Daejeon, 34943 Korea; 4 Department of Oral & Maxillofacial Surgery, Chungbuk National University Hospital, Chungdae-ro 1, Seowon-Gu, Cheongju, 28644 Chungbuk Korea; 5 Department of Oral & Maxillofacial Surgery, Hallym University Kangnam Sacred Heart Hospital, Singil-ro 1, Youngdeungpo-Gu, Seoul, 07441 Korea; 6 Department of Oral & Maxillofacial Surgery, Hankook General Hospital, Danjae-ro 106, Sangdang-Gu, Cheongju, 28713 Korea

**Keywords:** Denosumab, Denosumab-related osteonecrosis of the jaws, Necrosis of the jaws, Cancer, Anticancer drug

## Abstract

**Background:**

Denosumab (DMB) is a bone antiresorptive agent used to treat osteoporosis or metastatic cancer of the bones. However, denosumab-associated osteonecrosis of the jaw (DRONJ) has become a common complication in cancer patients. The prevalence of osteonecrosis of the jaw (ONJ) in cancer patients is estimated to be similar for both bisphosphonate-related cases (1.1 to 1.4%) and denosumab-related cases (0.8 to 2%), with the addition of adjunctive therapy with anti-angiogenic agents reportedly increasing its prevalence to 3%. (Spec Care Dentist 36(4):231–236, 2016). The aim of this study is to report on DRONJ in cancer patients treated with DMB (Xgeva®, 120mg).

**Case presentation:**

In this study, we identified four cases of ONJ among 74 patients receiving DMB therapy for metastatic cancer. Of the four patients, three had prostate cancer and one had breast cancer. Preceding tooth extraction within 2 months of the last DMB injection was found to be a risk factor for DRONJ. Pathological examination revealed that three patients had acute and chronic inflammation, including actinomycosis colonies. Among the four patients with DRONJ referred to us, three were successfully treated without complications and had no recurrence following surgical treatment, while one did not follow up. After healing, one patient experienced a recurrence at a different site. Sequestrectomy in conjunction with antibiotic therapy and cessation of DMB use proved to be effective in managing the condition, and the ONJ site healed after an average 5-month follow-up period.

**Conclusion:**

Conservative surgery, along with antibiotic therapy and discontinuation of DMB, was found to be effective in managing the condition. Additional studies are needed to investigate the contribution of steroids and anticancer drugs to jaw bone necrosis, the prevalence of multicenter cases, and whether there is any drug interaction with DMB.

## Background

The bone is the most common site of metastatic disease in advanced cancer, with skeletal metastasis occurring in nearly 100% of patients with myeloma, 65–80% with prostate or breast cancers, and 30–40% with lung cancer [1]. Bisphosphonates (BPs) are commonly used to treat and prevent skeletal problems associated with metastatic cancer. There is no doubt that BPs are helpful in treating cancer by preventing bone loss associated with chemotherapy, preventing hypercalcemia of malignancy, preventing cancer, and improving survival rates [2, 3]. However, osteonecrosis of the jaw (ONJ) can occur as a side effect in patients treated with BPs. For this reason, bisphosphonate-related osteonecrosis of the jaw (BRONJ) was first defined in 2007. The primary definition of BRONJ is a condition where there is exposed necrotic bone in the maxillofacial region that persists for more than 8 weeks in patients who have received or are receiving treatment with BPs and have no history of radiation therapy in the jaws [4].

Extensive research has been carried out with the aim of developing a medication that provides similar therapeutic benefits to BPs while minimizing the occurrence of side effects such as BRONJ. Consequently, Denosumab (DMB), a human monoclonal antibody that effectively inhibits the activity of osteoclasts, has been identified as a successful outcome of these efforts [5]. DMB has been shown to have an equal or greater capacity to suppress bone turnover than bisphosphonates [6]. DMB is different from BPs in that it is designed to inhibit the receptor activator of nuclear factor kappa-B ligand (RANKL), which is a protein that serves as the main signal for bone resorption [7]. However, clinicians are now observing denosumab-related osteonecrosis of the jaws (DRONJ), which has a similar clinical presentation to BRONJ [8]. Anti-resorptive medication is a major risk factor for causing medication-related osteonecrosis of the jaw (MRONJ) directly in cancer patients.

Recently, antiangiogenic drugs like sunitinib, sorafenib, and bevacizumab have been associated with the development of ONJ, similar to that induced by BPs. Anti-angiogenic drugs are one of the risk factors that can cause ONJ, and an increasing number of anti-angiogenic medications have been shown to cause medication-related osteonecrosis of the jaw (MRONJ).

Although the incidence of MRONJ has increased in recent years, there have been few reports of DRONJ or anticancer drugs. In osteoporosis treatment, a low dose of 60 mg DMB is typically given every 6 months, while a high dose of 120 mg DMB is given monthly for cancer treatment. Due to this difference, a separate study is needed to examine DRONJ in cancer patients receiving high-dose DMB, but there are currently limited studies on this topic. Furthermore, reported cases of ONJ related to the combination therapy of DMB and anticancer drugs are not frequent enough to establish an effective treatment plan. In this report, we present a case of MRONJ in cancer patients with bone metastasis receiving combination therapy of DMB and anticancer drugs. And we aim to report on the cases of patients we have successfully treated with surgery, along with a review of the literature.

## Methods

We analyzed the medical records of 74 patients treated with DMB (Xgeva 120mg, Amgen, Thousand Oaks, California, USA) for metastatic cancer at 00 University Hospital from January 2016 to October 2021. Patients who had received orofacial radiotherapy or previous treatment with BPs were excluded.

## Results

Of the 74 patients, four developed DRONJ. Three of the four patients had prostate cancer, while the remaining patient had breast cancer. All of the patients were taking anticancer drugs, specifically Docetaxel and Trastuzumab. One case of DRONJ occurred in a patient who did not receive any dental treatment, while the other cases were associated with tooth extraction within 2 months of the last DMB injection. As a pathological diagnosis, three patients were found to have acute and chronic inflammation, including colonies of actinomyces. Three of the four patients with DRONJ were successfully treated through surgical intervention without any complications or recurrence. Unfortunately, the other patient was not followed up. After the initial healing, one patient experienced another occurrence of DRONJ at a different site. However, the condition was effectively managed through sequestrectomy, antibiotic therapy using Augmentin (amoxicillin and clavulanic acid), and discontinuation of DMB use. After an average of 5 months of follow-up, the ONJ site was fully healed (Table 1).

**Table 1:** Clinical descriptions of the patients with DRONJ

	Patient 1	Patient 2	Patient 3	Patient 4
Sex	Male	Male	Female	Male
Age	65	75	79	82
Primary cancer	Prostate	Prostate	Breast	Prostate
Concomitant treatment	Docetaxel Corticosteroids	Docetaxel Enzalutamide Corticosteroids	Trastuzumab	Docetaxel Corticosteroids
ONJ-related underlying diseases	-	Diabetes	Diabetes Rheumatoid arthritis	-
ONJ site	Mandible posterior	Mandible posterior	Mandible anterior	Maxilla anterior
Cause of onset	Tooth extraction	Tooth extraction	Spontaneous onset	Tooth extraction, denture
Number of injections at the time of DRONJ diagnosis	13	10	12	1
A cycle of DMB injection (month)	1	1	1.5	-
Duration of the DMB treatment before onset (month)	11	6	19	2
Interval between extraction and last DMB injection (month)	1	1	-	2
Duration of drug holiday before the ONJ surgery (month)	6	1	4.5	-
Healing period after ONJ surgery (month)	2	2	1st surgery: 5 2nd surgery: 5	^a^
Actinomyces finding	Y	Y	Y	-
DRONJ status at the end of follow up	Stabilization	Stabilization	Stabilization	Unknown/death

^a^No follow-up

## Case presentation

### Case 1: 120 mg DMB, docetaxel

**Fig. 1:**
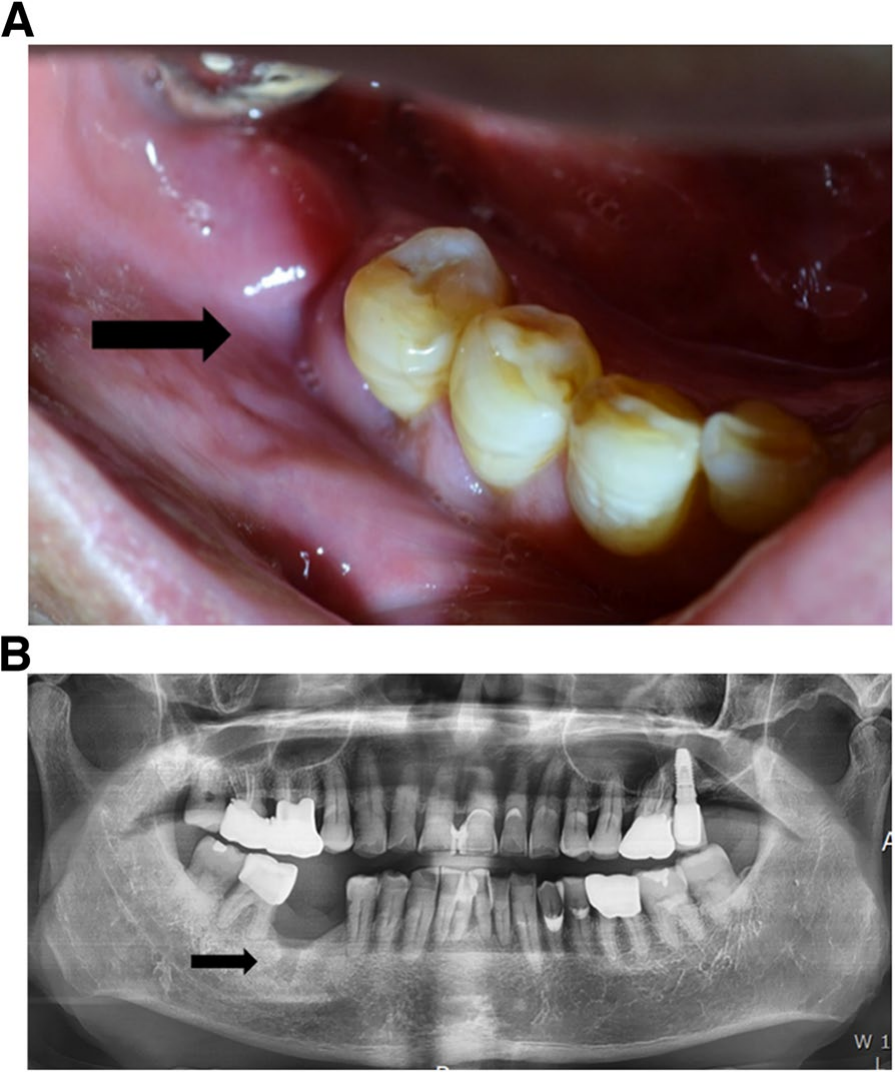
Non-healed state of the spontaneous removal site of #46 implant. **A** Intraoral photo. Inflammatory state on the spontaneous removal implant. **B** A panoramic view on the first visit. Non-healed state of the removal site of #46 implant

A 65-year-old male patient was referred to our department by his urologist due to the development of DRONJ within 13 months of starting DMB. The DMB regimen consisted of monthly subcutaneous injections of 120 mg DMB. The patient had metastatic prostate cancer that was no longer responding to hormone therapy. However, he did not have a history of receiving BPs or radiotherapy to the head and neck region. The DRONJ region was first noticed about 11 months after starting DMB, and it was located at the site where the #46 implant was spontaneously removed. The patient’s local dentist reported a previous infection and a non-healed state in this area (Fig. 1A, B). He was referred to our hospital because the bone in the DRONJ region did not heal even after 2 months of removing the implant. At this stage, his urologist had stopped the monthly Xgeva® injections due to the DRONJ; he had received a total of thirteen doses.

**Fig. 2:**
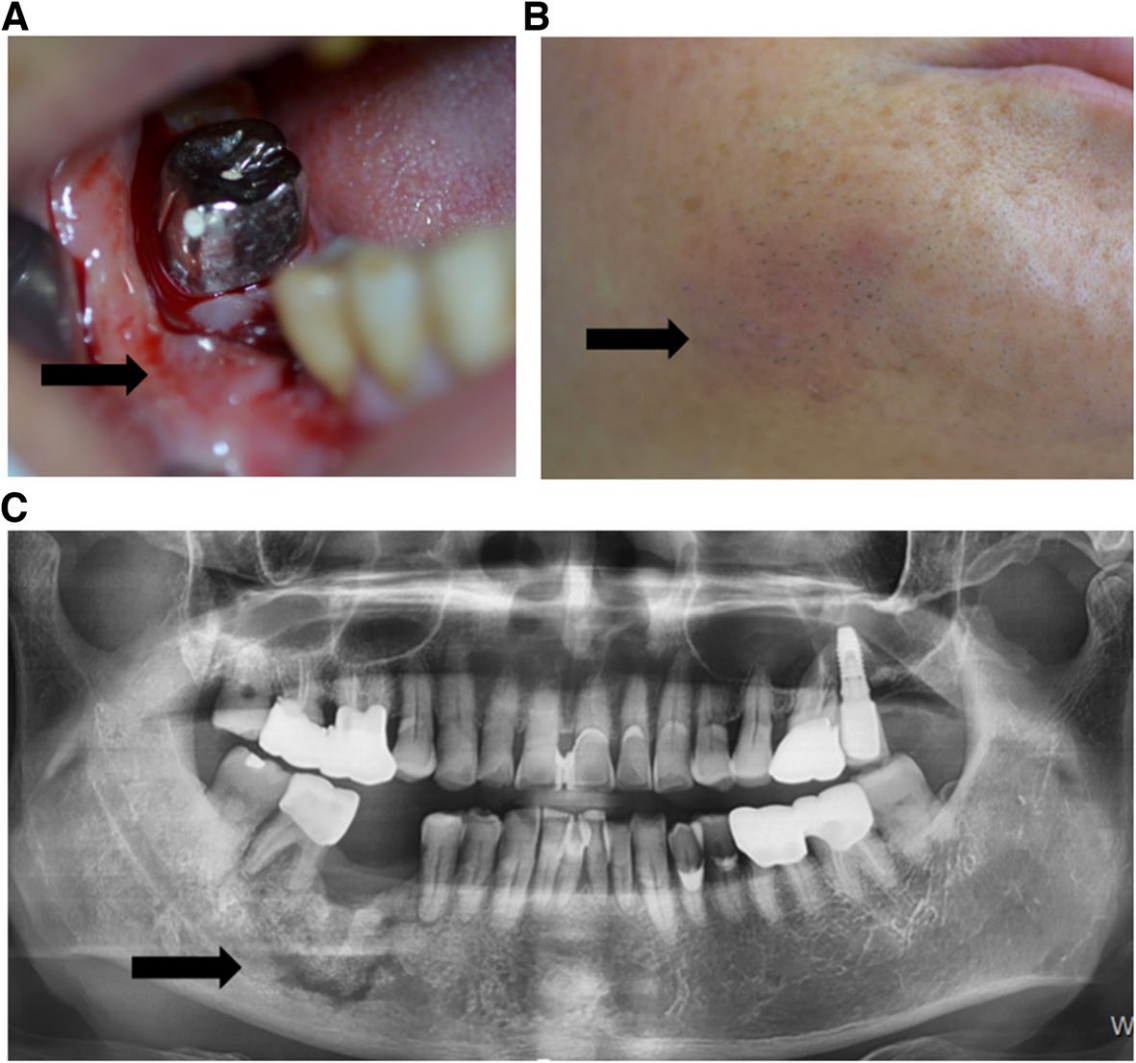
Preoperative state. After 4 months of conservative treatment. **A** Intraoral photo. **B** Facial photo. Erythematous and inflamed in the right buccal cheek area. **C** A panoramic view. Sequestra formation on Rt. mandibular posterior area

**Fig. 3:**
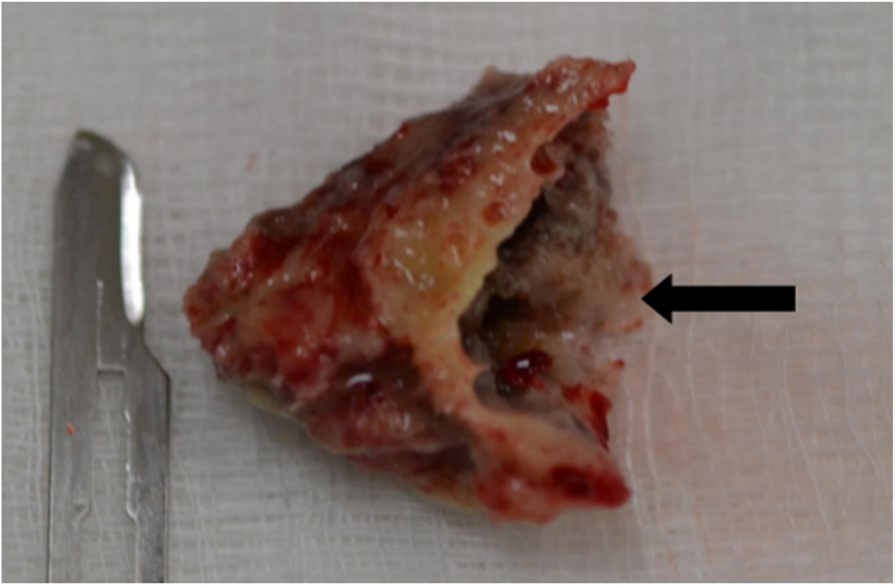
Necrotic bone fragment

**Fig. 4:**
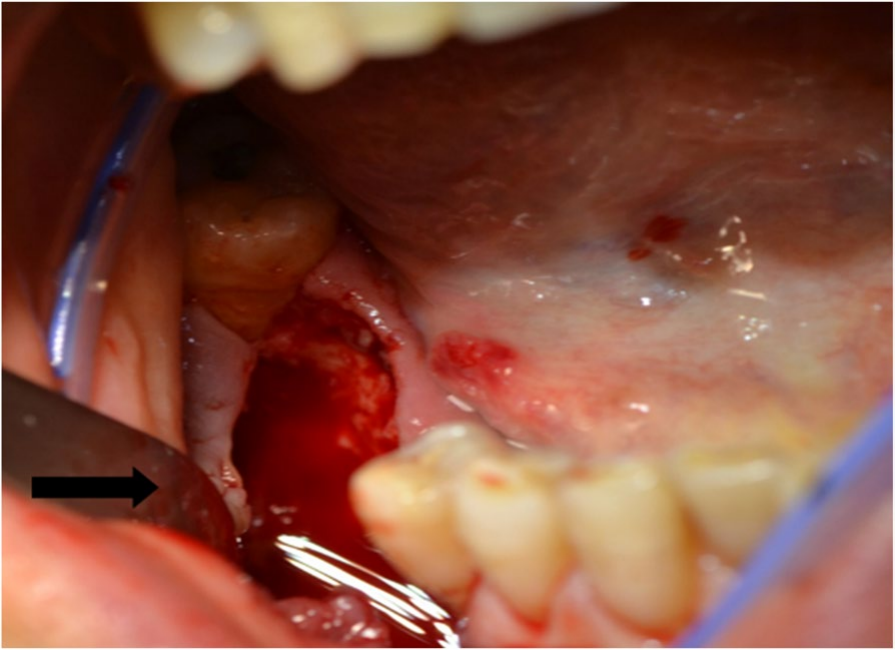
Intraoperative photo of debridement

**Fig. 5:**
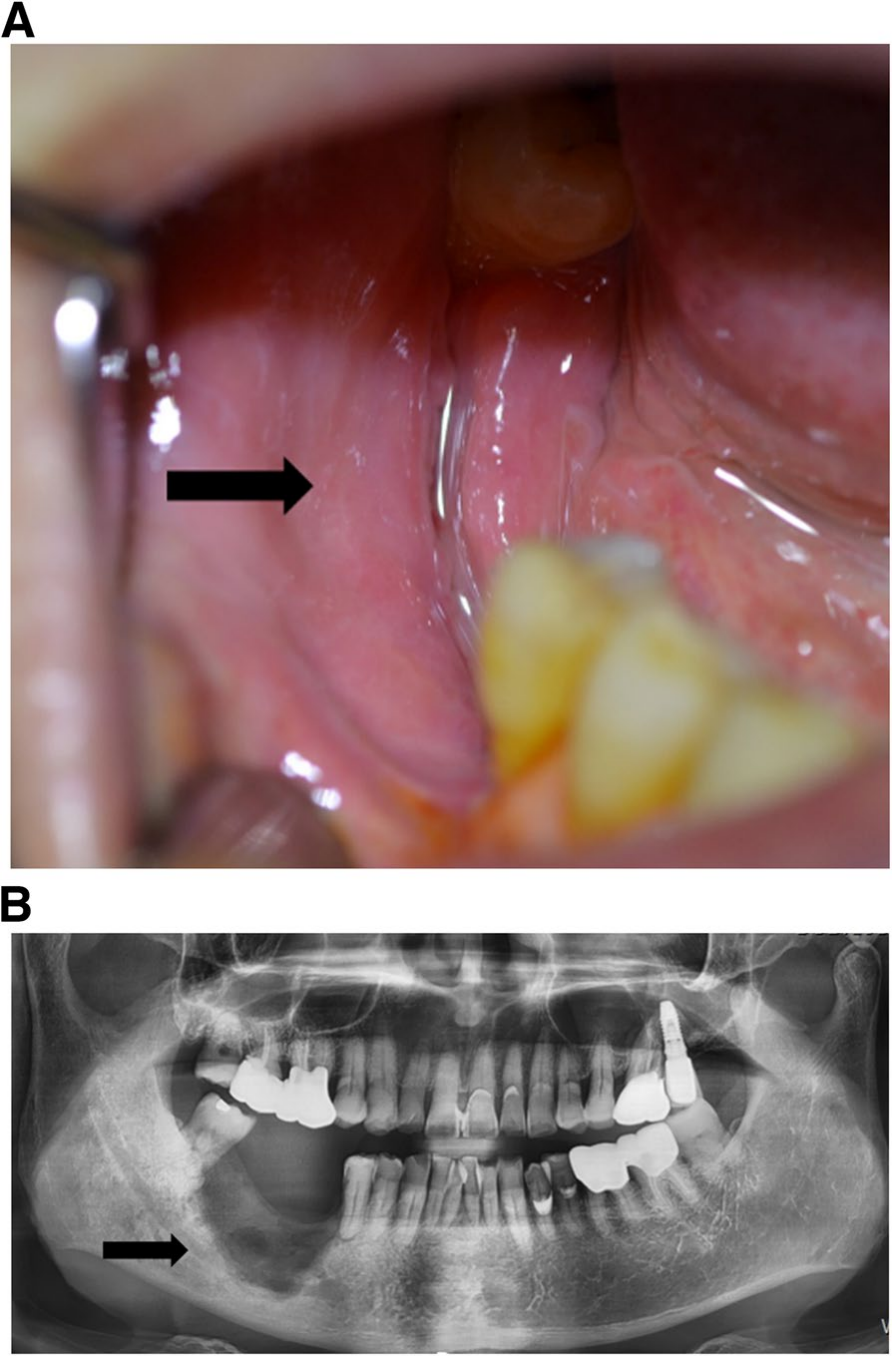
Postoperative 2 months. **A** Complete closure state of the intraoral oral area. **B** Panoramic view. It showed the defect has not been fully closed to date

Conservative treatment was started initially with antibiotics, chlorhexidine mouth rinses, and oral hygiene advice. However, after 4 months of conservative treatment, the area around the removal site of the implant and the surrounding soft tissues, including tooth #47, were still inflamed and erythematous (Fig. 2A, B, C). Despite undergoing conservative treatment, ONJ did not improve and the patient’s clinical stage of MRONJ progressed to stage 2. Therefore, we planned to perform a surgical treatment for ONJ. Under local anesthesia, a necrotic bone fragment measuring 12 mm × 10 mm × 5 mm was removed 6 months after discontinuing DMB (Fig. 3). The affected area was superficially debrided to ensure that healthy and vital bone remained at the base of the region. The wound was then closed using primary closure techniques (Fig. 4). A sequestrum from the non-healing socket area with the exposed bone was sent for histopathological examination, and the results confirmed the presence of non-vital bone and actinomycotic infection. The defect size has decreased, and conservative measures have helped to maintain healthy soft tissues in the area. However, the defect had not yet completely healed. The patient was followed up clinically and radiographically for 2 months, and at the end of this period, the intraoral area showed complete closure (Fig. 5A, B). No pathologic fracture or DRONJ recurrence was observed.

### Case 2: 120 mg DMB, docetaxel

**Fig. 6:**
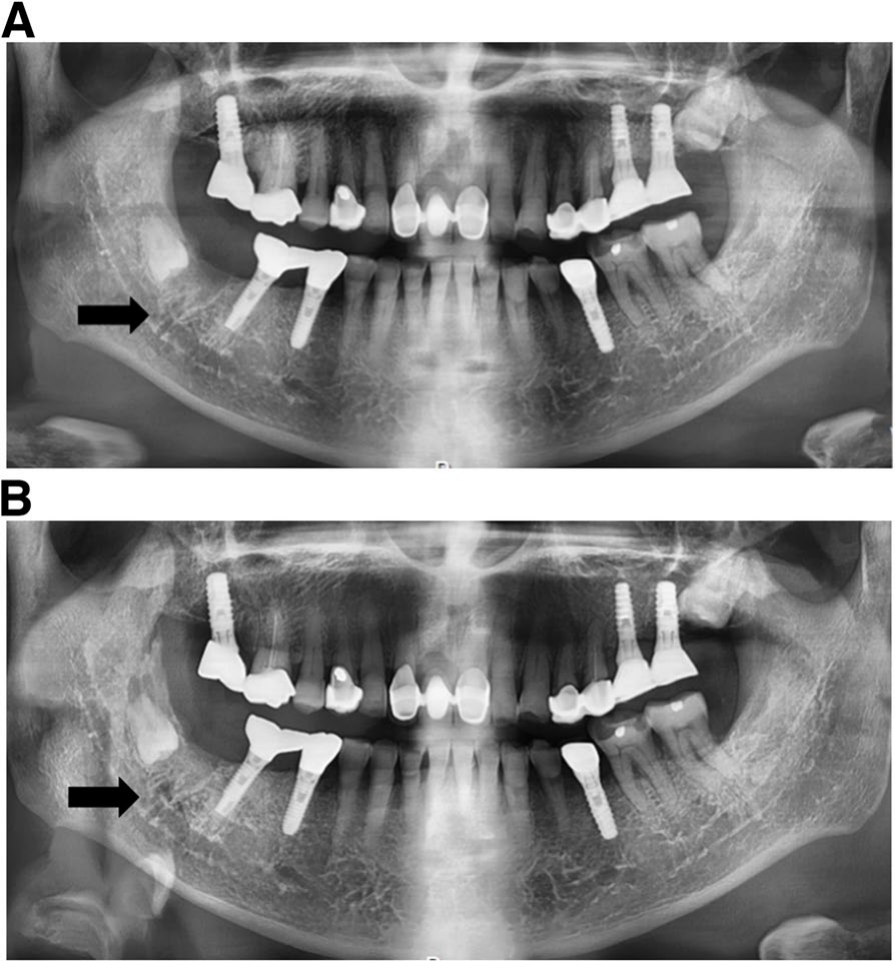
**A** Panoramic view on the first visit. Alveolar bone inflammation was observed in the socket of tooth #47. **B** A 7-month postoperative panoramic view. Signs of bony infill were observed at the site of the #47 socket

A 75-year-old male was referred to our clinic by his general dental practitioner due to constant pain and recurrent infection in the socket of tooth #47, which was extracted 3 months ago. During the examination, a gingival swelling was observed in the lower right molar area, accompanied by purulent discharge. In the panoramic view, evidence of alveolar bone inflammation was observed in the socket of tooth #47 (Fig. 6A). The patient had a medical history of prostate cancer, diagnosed in 2018, and had been receiving intravenous DMB injections for metastatic disease since 2019. Before starting monthly DMB injections, the patient had been taking steroids and anti-cancer drugs, specifically enzalutamide and docetaxel. He was also being treated for diabetes. The 120mg DMB injections were administered every 4 weeks for a duration of 10 months. There was no history of previous radiotherapy or other anti-resorptive treatments noted.

Upon clinical examination, osteomyelitis and gingival swelling were observed at the site where tooth #47 had been extracted. As the patient’s stage of MRONJ was determined to be at stage 2 and did not improve despite long-term antibiotic therapy, debridement was planned. Additionally, in consultation with his oncologist, it was decided to discontinue the use of DMB. Approximately 1 month after the last DMB injection, surgical curettage was performed, along with the removal of the sequestrum. The sequestrum was sent for histopathological examination, which confirmed the presence of necrotic bone and actinomycotic infection. The lesion was treated conservatively with long-term antibiotic coverage and proper oral hygiene practices. At the 2-month postoperative follow-up appointment, significant clinical improvement and gingival wound healing were observed. Additionally, at the 7-month postoperative review, signs of bony infill were observed at the site of the #47 socket following the sequestrectomy (Fig. 6B).

### Case 3: 120 mg DMB, trastuzumab

A 79-year-old female was referred to our oral surgery department with persistent pain at the root rest of the #33 tooth site. The patient was under the care of her oncologist for breast cancer, which had been resected 20 years ago. She received chemotherapy approximately 1 year ago and had been taking the anti-cancer drug trastuzumab for 1 year. She was also diagnosed with diabetes and rheumatoid arthritis. DMB was administered in the form of 120mg injections every 6 weeks for the past one and a half years after receiving chemotherapy. She had no previous history of radiotherapy or other anti-resorptive treatments.

**Fig. 7:**
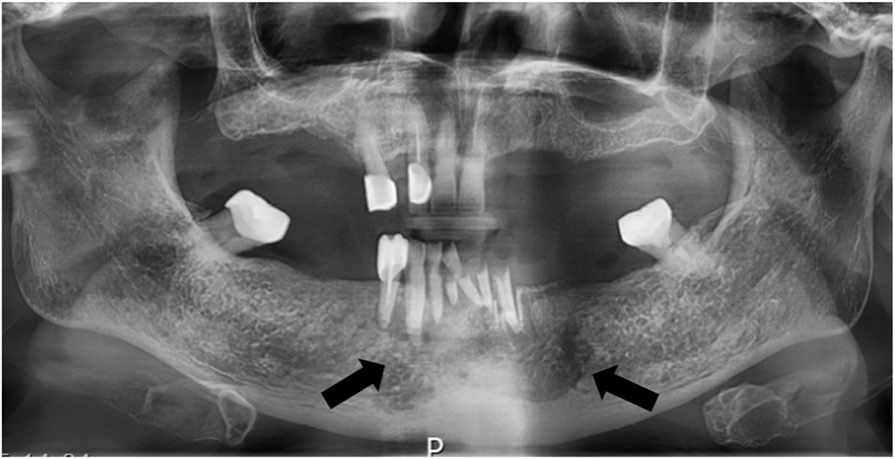
Panoramic radiograph on the first visit. Radiolucent periapical lesion around the #41-33 region and ill-defined bone loss

On her initial visit, a panoramic radiograph showed a radiolucent periapical lesion around the #41-33 region and ill-defined bone loss (Fig. 7). When the patient first visited the oral surgery department, she had already discontinued DMB injections for 4 months. One week later, surgical removal of the necrotic bone fragment on the posterior mandibular area was performed along with the extraction of teeth #31, 32, 33, 41, 42, 43, and 44, local debridement, and biopsy of the necrotic tissue. The histological examination showed osteonecrosis of the jaw with actinomycosis.

**Fig. 8:**
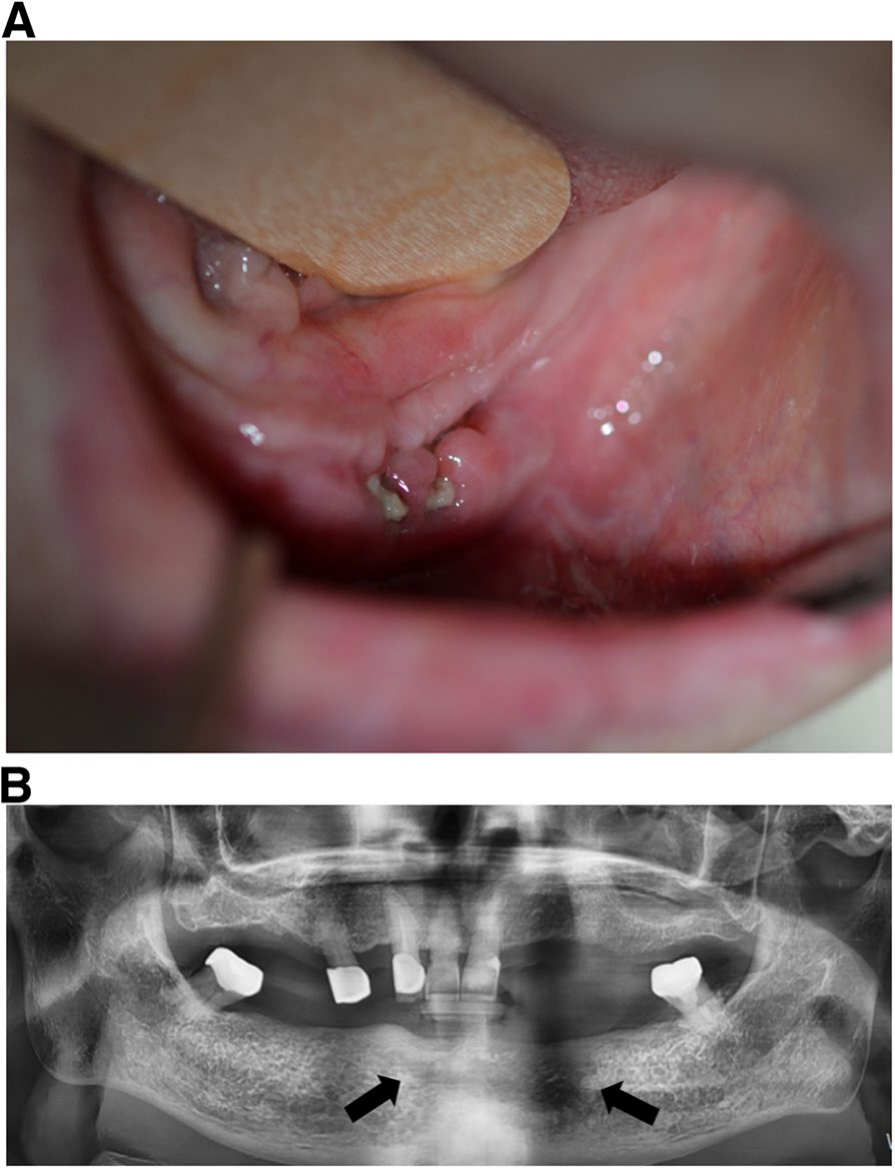
Postoperative 5 months. **A** Mild gingival swelling and bone exposure in the left posterior mandibular region. **B** Panoramic radiograph. Radiolucent periapical lesion in the left posterior mandibular region

At the 5-month postoperative follow-up, the anterior mandibular area was healed. However, 1 month later, mild gingival swelling and bone exposure were observed in the area of the left mandibular premolar where the teeth were missing (Fig. 8A). A panoramic radiograph revealed the presence of a radiolucent periapical lesion in that same region (Fig. 8B). Based on the clinical and radiographic findings, the patient underwent a second surgical treatment for recurrent ONJ under local anesthesia. The surgical procedure involved the complete removal of the necrotic bone and infected soft tissues. At the 5-month follow-up after the second surgery, complete healing was observed in the treated area.

### Case 4: 120 mg DMB, docetaxel

An 82-year-old male with metastatic prostate cancer was referred to our clinic by his dentist. He had been experiencing persistent pain in the right anterior maxilla since having a tooth extraction 2 months ago. The patient was recently given a new temporary full denture for the maxilla after the upper teeth extraction, but it has been causing discomfort. He underwent chemotherapy 1 year ago and prostate radiation therapy 3 months prior to his first dental visit. At the time of his visit, he was taking the anti-cancer drug docetaxel and had only received one injection of 120mg DMB. The time between the tooth extraction and the DMB injection was only 2 months. The patient had not been prescribed bisphosphonates and had not undergone radiation therapy in the head and neck area.

**Fig. 9:**
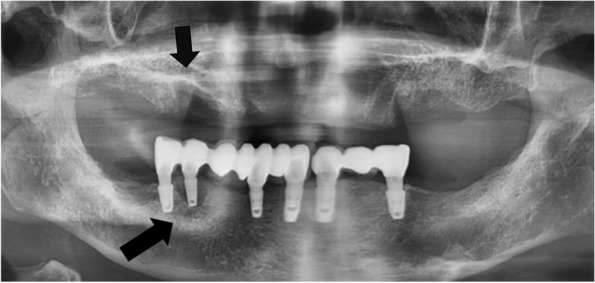
Panoramic radiograph. Osteomyelitis around the socket of the right anterior maxilla after tooth extraction, as well as a radiolucent lesion below the #44 implant

The panoramic radiograph revealed osteomyelitis around the socket of the right anterior maxilla after tooth extraction, as well as a radiolucent lesion below the #44 implant (Fig. 9). Based on these clinical and radiological findings, the presumptive diagnosis was DRONJ. We requested the patient’s oncologist to discontinue DMB prescription and implemented oral chlorhexidine irrigation, as well as inner adjustments to the temporary denture. Conservative surgery for ONJ was planned, which involved extracting the affected area in the maxilla and removing the #44 implant. However, we were unable to provide follow-up care or treatment as the patient did not return to the hospital.

## Discussion

BPs are commonly used for the prevention and treatment of skeletal-related events that are linked to both metastatic cancer and osteoporosis. It is undeniable that BPs can enhance the quality of life, prevent bone loss during chemotherapy, prevent hypercalcemia associated with malignancy, and aid in the prevention of cancer. Despite their benefits, BPs pose a risk of BRONJ for patients who receive them. Therefore, it became necessary to develop a drug that can provide the same therapeutic benefits as BPs without the risk of side effects such as ONJ. This has led to the discovery of DMB, which is a human monoclonal antibody that can impede osteoclast function [4]. In June 2010, the US Food and Drug Administration (FDA) approved the use of DMB, marketed under the trade name Prolia® (Amgen in Thousand Oaks, California, USA). This particular formulation is intended for use in postmenopausal women at risk of osteoporosis, with the standard regimen being a subcutaneous injection of 60 mg DMB every six months. In November 2010, the FDA approved DMB in the form of Xgeva® (Amgen, Thousand Oaks, California, USA), for the prevention of skeletal-related events in patients with bone metastasis from solid tumors [4].

With the increasing number of patients taking DMB, clinicians have started observing denosumab-associated osteonecrosis of the jaws (DONJ), which shows a similar clinical presentation to BRONJ. There are several hypotheses aimed at explaining the unique localization of ONJ when related to systemic anti-resorptive medications. The literature suggests similar mechanisms for the pathophysiology of DRONJ and BRONJ [9]. These theories include altered bone remodeling, inhibition of angiogenesis, repetitive and persistent microtrauma, impaired tissue protection and soft tissue anatomy, toxicity of BPs, and bacterial infection [10, 11].

However, DMB and BPs have different mechanisms of action, and it has been reported that the pharmacokinetics of DMB are more favorable in managing ONJ than those of BPs. The main factor contributing to this difference is that BPs, once absorbed, have a half-life of approximately 10 years, whereas the interaction between DMB and bone is more reversible [12]. DMB is a fully human monoclonal immunoglobulin 2G (Ig2G) antibody that works by inhibiting a critical step in the differentiation of osteoclasts, which leads to a decrease in bone metabolism. Specifically, DMB blocks the binding of RANKL to its receptor, receptor activator of nuclear factor kappa (RANK), which is located in the cell membranes of both osteoclasts and osteoclast precursor cells. Pharmacologically, DMB acts by mimicking the function of the endogenous molecule osteoprotegerin (OPG). RANK, RANKL, and OPG are all members of the tumor necrosis factor superfamily of proteins with OPG having a particularly strong antiresorptive effect. OPG has a significant impact on the regulation and balance of osteogenesis by inhibiting the formation, attachment, and activation of osteoclasts, as well as increasing osteoclast apoptosis [13]. Working together, RANKL and OPG maintain a healthy balance of bone resorption. However, if the RANKL/OPG ratio increases, it can tip the balance in favor of bone resorption, leading to skeletal diseases [14].

Unlike bisphosphonates, DMB circulates in the bloodstream and does not accumulate in bone tissue. This is due to differences in their pharmacological mechanisms. While the elimination half-life of DMB is much shorter (26 days) than that of bisphosphonates (10 years), its pharmacodynamic half-life, which refers to the duration of its antiresorptive effect, is longer than its elimination half-life [15]. A study by Bone et al. found that it took 9 months for the antiresorptive effects of a 60-mg dose of DMB for osteoporosis to fully reverse. Based on this, it can be assumed that the antiresorptive effects of a 120-mg dose of DMB would last for at least 9 months [16]. However, for most oncology patients, DMB is a life-prolonging drug that cannot be discontinued for such a long period.

Despite the potential of DMB to cause osteonecrosis due to its mechanism of action, the summary of product characteristics for Xgeva® (DMB 120 mg) does not indicate that MRONJ occurrence is a contraindication for reintroducing the drug [17]. Normally, DMB is administered in two dosage schemes: 60 mg subcutaneously every 6 months for the treatment of osteoporosis or 120 mg subcutaneously monthly for the prevention of skeletal-related events in cancer patients. In this study, all four cancer patients received DMB 120mg (Xgeva®) every 4 to 6 weeks.

The prevalence of ONJ in cancer patients is estimated to be similar for both BP-related cases (1.1 to 1.4%) and DMB-related cases (0.8 to 2%). The prevalence may increase to 3% in patients receiving adjunctive therapy with anti-angiogenic agents, especially in renal cell carcinoma [8]. According to the literature, the incidence of MRONJ in cancer patients treated with DMB 120 mg ranges from 0.7 to 11.4% [17].

In this study, we observed four cases of ONJ among 74 patients who received DMB therapy for metastatic cancer. Among the four patients who developed ONJ, three had prostate cancer, while the remaining patient had a history of breast cancer. The patients with prostate cancer were being treated with docetaxel and enzalutamide, whereas the patient with breast cancer was receiving trastuzumab. Furthermore, three out of the four cancer patients were also taking corticosteroids.

Studies have reported that ONJ induced by BP usually occurs after approximately 39.3 months and 35 infusions in oncology patients. However, it is interesting to note that all reported cases of ONJ related to DMB occurred early in the course of DMB therapy, regardless of the number of prior administrations [18]. In these reported cases, the average number of DMB injections was 8 and ONJ occurred within 1 year of starting the therapy (Table 1).

The relationship between anti-angiogenic medications and osteonecrosis of the mandible has been well established, and this is believed to be the underlying cause in cancer patients. The risk of ONJ may be increased when anti-angiogenic agents and steroids are used concomitantly with anti-resorptive agents, which is becoming more common in clinical practice [19].

Trastuzumab, a monoclonal antibody that inhibits epidermal growth factor, is an anti-angiogenic agent that is commonly used in the treatment of breast cancer. Previous studies have identified it as an independent risk factor for the development of ONJ. In some cases, ONJ has been reported to occur during concurrent treatment with bisphosphonates and trastuzumab [20]. Additionally, there have been case reports in the recent literature of ONJ developing solely due to trastuzumab treatment [21]. However, an association of trastuzumab with the occurrence of ONJ has not been clearly stated in these reports.

The anti-neoplastic agent TAX is frequently used in the treatment of prostate cancer. However, the present findings suggest that leukopenia induced by TAX is more relevant than TAX use itself in the development of ONJ [22]. The anti-angiogenic properties of both DMB and TAX may have an additive or synergistic effect on impairing jaw vascularization, leading to a higher susceptibility to bone necrosis.

While the exact cause of osteonecrosis in patients receiving a combination of DMB and anti-cancer drugs is not clear, it is believed that the incidence may be higher due to the presence of anti-angiogenic agents and steroids, which are known risk factors for osteonecrosis. Further research is necessary to better understand the underlying mechanisms and potential preventive measures.

Actinomycosis was diagnosed in three out of four patients in this case report through pathological examination. The presumed cause of the actinomycete infection at the site of osteonecrosis is the immunosuppressive effect of anticancer drugs and steroids. By blocking RANKL activity, DMB can cause a decrease in macrophage and monocyte function and survival, which can ultimately impact the immune system and result in immunosuppression [23]. And this immunosuppressive effect would have resulted in an environment prone to infection with actinomycetes.

DRONJ is generally triggered by surgical manipulation of the affected area, such as dental extractions or implant placements. In addition to surgical manipulation, other factors such as trauma from ill-fitting removable partial dentures or full dentures, and impaction of debris under pontics, have also been reported as predisposing factors for DRONJ [24]. In this report, the main cause of ONJ was identified as surgical tooth extraction. These patients had undergone dental extractions within 2 months of their most recent DMB injection, which resulted in the induction of osteonecrosis. The recently published dental guidelines recommend that patients who are receiving a high dose of DMB (120mg Xgeva®) should receive any necessary surgical procedures at least 3 weeks after their last administration of the drug [25]. However, ONJ was still induced even though tooth extraction was performed 3 weeks after the last DMB administration. Furthermore, Hasegawa et al. have reported that a 1-month washout period before tooth extraction in patients with cancer who were receiving high-dose DMB did not reduce the incidence of MRONJ [26]. Therefore, further research is needed as there is no established guideline for minimizing the occurrence of MRONJ before dental surgical intervention in cancer patients treated with high-dose DMB. Additionally, preventive dental treatment is required before administration of DMB, as it has been found that underlying oral diseases such as periodontitis and peri-implantitis can induce ONJ [27].

In case 3, the patient developed DRONJ without surgical intervention. It is assumed that inflammation in the residual root caused the ONJ. Pre-existing periodontal disease or periapical pathology has been cited as a risk factor of MRONJ. Pre-existing inflammatory dental disease has been found to be a risk factor for MRONJ in 50% of cancer patients with the condition [27]. Therefore, it is recommended to consider periodontal disease treatment or tooth extraction prior to the administration of anticancer drugs in cancer patients to prevent MRONJ.

In this study, patients with ONJ were treated with sequestrectomy and antibiotic therapy using Augmentin. Sequestrectomy is a surgical procedure that involves removing a section of the necrotic bone that has become separated from the surrounding healthy bone. Minimally invasive surgical techniques are considered the most effective treatment for early stages (I and II) of MRONJ [28]. Among minimally invasive surgical options, debridement and sequestrectomy are commonly used. Debridement involves surgically removing dead bone tissue until healthy, bleeding bone is exposed and is typically performed when some healthy bone is still present.

Currently, there is limited understanding of the pathogenic mechanism of jawbone necrosis related to DMB treatment, and there is a lack of conclusive evidence on the effectiveness of drug suspension as a preventive measure, as well as the appropriate timing for this approach [27]. Recently, there has been a suggestion that performing oral surgery during a protective period of 5 to 7 months between the last and next dose of DMB may be a reasonable approach pharmacokinetically [29]. Regardless of the stage of the disease, cancer patients generally require more extensive surgical procedures due to the more severe progression of the disease compared to osteoporotic patients [30]. For this reason, in the case presented, the bone involvement (stage 2 ONJ) provided the indication for the debridement surgery of the necrotic bone, following the treatment guidelines based on the MRONJ staging system [27]. However, there is still a lack of conclusive evidence regarding the optimal suspension period for high-dose DMB and the appropriate timing of oral surgery in cancer patients.

For the first patient, who presented with stage 0-1 MRONJ, antibacterial oral rinse therapy was administered. However, after 4 months of treatment, the MRONJ did not improve and progressed to stage 2, indicating the necessity of surgical treatment. For the second patient, who presented with stage 2 MRONJ and did not show improvement despite over three months of antibiotic therapy, discontinuation of DMB was advised, and immediate surgical intervention was deemed necessary. As a result, surgical debridement was performed on the patient within a month of discontinuing DMB. The third patient presented with a mandibular sequestrum and had stopped using DMB 4 months prior to the visit. Consequently, a surgical plan was established on the first day of the visit, and a sequestrectomy was performed within a month.

The surgical site healed in less than 4 months for most patients. However, one patient developed postoperative ONJ at another site after 5 months and required an additional operation. It took a total of 10 months for this patient’s ONJ to fully heal.

## Conclusion

This study indicates that dentists should exercise more caution when providing treatment to cancer patients receiving high doses of DMB injections and chemotherapy. Dental prescreening prior to DMB administration is necessary to prevent DRONJ. A careful surgical approach, along with the use of antibiotics and discontinuation of DMB after consultation with the patient’s medical doctor, appears to be an effective strategy for managing DRONJ. For stage II ONJ by high-dose denosumab, immediate minimally invasive surgical intervention (sequestrectomy, debridement) and discontinuation of denosumab were effective for treatment.

Several types of anti-angiogenic drugs are utilized in the treatment of cancer; however, the specific mechanism by which each drug induces osteonecrosis is not well understood. It is also unclear whether there is an interaction between anti-cancer drugs and DMB that contributes to ONJ in cancer patients when used in combination. Therefore, additional research is necessary to establish dental treatment guidelines for cancer patients who receive both DMB and anticancer drugs.

## Data Availability

The datasets used and/or analyzed during the current study are available from the corresponding author on reasonable request.

## Abbreviations

BP: Bisphosphonate
BRONJ: Bisphosphonate-related osteonecrosis of the jaw
CBCT: Cone beam computed tomography
DMB: Denosumab
DONJ: Denosumab-associated osteonecrosis of the jaws
DRONJ: Denosumab-related osteonecrosis of the jaw
MRONJ: Medication-related osteonecrosis of the jaw
ONJ: Osteonecrosis of the jaws
OPG: Osteoprotegerin
